# Adverse effects associated with peribulbar injection of triamcinolone for the treatment of thyroid eye disease: a retrospective case series

**DOI:** 10.5935/0004-2749.20230063

**Published:** 2023

**Authors:** Joaquin Miguel Gonzalez Barlatay, Carla Pagano Boza, Tomaz Ortiz Basso, Mercedes Benavente, Guillermo Vicente Hernández-Gauna, Eduardo Jorge Premoli

**Affiliations:** 1 Division of Orbital and Ophthalmic Plastic Surgery, Hospital Italiano de Buenos Aires, Buenos Aires, Argentina.

**Keywords:** Orbit/diagnostic imaging, Magnetic resonance imaging, Graves ophthalmopathy, Triamcinolone/adverse effects, Injections, Órbita/diagnóstico por imagem, Imageamento por ressonância magnética, Oftalmopatia de Graves, Triancinolona/efeitos adversos, Injeções

## Abstract

**Purpose:**

Peribulbar injection of triamcinolone is an alternative treatment for thyroid eye disease; however the safety profile of this therapeutic option remains controversial. The aim of this study was to describe the occurrence of local and systemic adverse effects after peribulbar injection of triamcinolone in patients with thyroid eye disease.

**Methods:**

This was a retrospective case series. Medical records of patients with thyroid eye disease treated with peribulbar injections of triamcinolone at a single academic institution between 2007 and 2019 were analyzed. Local and systemic complications were documented.

**Results:**

A total of 123 patients were treated. Only 11 patients (8.9%) developed local complications. The most frequent complication was the presence of superficial eyelid ecchymosis (nine patients; 7.3%). Notably, systemic complications (hyperglycemic and suprarenal inhibition after stop treatment) occurred in two patients (1.6%). All complications were transient, and the patients did not have any long-term sequelae.

**Conclusions:**

Peribulbar injection of triamcinolone for the treatment of thyroid eye disease is linked to a very low rate of local or systemic complications. Prospective studies are warranted to delve into this topic.

## Abstract (pt)

**Objetivo:**

A injeção peribulbar de triancinolona é um tratamento alternativo para doenças oculares da tireoide; no entanto, a segurança desse procedimento continua controversa. O objetivo deste artigo é descrever os efeitos adversos locais e sistêmicos de injeções peribulbares de triancinolona em pacientes com doença ocular da tireoide.

**Métodos:**

Estudo retrospectivo de uma série de casos. Foram analisados os prontuários médicos dos pacientes com doença ocular da tireoide tratados com injeções de triancinolona peribulbar em uma única instituição acadêmica entre 2007 e 2019. Foram documentadas as complicações locais e sistêmicas.

**Resultados:**

Um total de 123 pacientes foram tratados. Apenas 11 (8,9%) pacientes apresentaram complicações locais, sendo a mais frequente a presença de equimoses palpebrais superficiais (7,3%), enquanto 2 (1,6%) pacientes apresentaram complicações sistêmicas (hiperglicemia e inibição da suprarrenal após a interrupção do tratamento). Todas estas complicações foram transitórias e nenhum paciente apresentou sequelas de longo prazo. **Conclusões:** As injeções peribulbares de triancinolona nas doenças oculares da tireoide têm uma taxa muito baixa de complicações, tanto locais quanto sistêmicas. São necessários estudos prospectivos para aprofundar este tópico.

## INTRODUCTION

Thyroid eye disease (TED) is an autoimmune disorder characterized by mild, moderate, or severe inflammation of the orbital tissues, and may even lead to visual impairment. Orbital changes in TED are the consequence of *de novo* adipogenesis, hyaluronan synthesis, interstitial edema, and enlargement of extraocular muscles under active and transitory orbital inflammation. Orbital fibroblasts express both thyroid stimulating hormone receptor and insulin-like growth factor-1 receptor (IGF-1R) at higher levels than normal fibroblasts, causing adipogenesis and production of hyaluronan. IGF-1R stimulation leads to adipogenesis, hyaluronan synthesis, and production of chemokines activating and increasing the number of T cells into the orbit. Cytokines, including interleukin-1β (IL-1β), IL-1α, IL-6, and IL-8, and macrophages perpetuate the orbital inflammation^(1)^. The gold standard treatment for the active phase of TED is intravenous systemic corticosteroids. The efficacy and appropriate dosage of this treatment have been demonstrated through controlled and randomized clinical trials, and analyzed in systematic reviews^(2-6)^. Due to the established and wide variety of adverse effects, in recent years, different options of treatment were investigated. These include diverse routes fort the administration of steroids (peribulbar injections) and other types of drugs, such as monoclonal antibodies (tocilizumab, teprotumumab). Evidence from randomized clinical trials has shown that both tocilizumab and teprotumumab offer a clinically significant improvement in activity and severity, along with a low rate of adverse events. Further studies are warranted to characterize the role of these drugs in TED. Notably, the cost of these drugs is markedly higher compared with that of corticosteroids^(7-10)^.

In 2004, Ebner et al.^(11)^ conducted a prospective and multicenter study, describing for the first time the advantages of the administration of peribulbar triamcinolone (PT) in patients with TED. This route of administration results in local anti-inflammatory effects on the affected tissues, thereby reducing the systemic exposure to corticosteroids and possibility of side effects. The investigators demonstrated that PT reduces the size of extraocular muscles and diplopia in patients with newly diagnosed TED. In addition, clinical complications related to this treatment route were not recorded in that study.

Subsequently, numerous researchers reported the benefits of PT in TED^(12-16)^. Moreover, in 2009, Bordaberry et al.^(12)^ reported that PT injections reversed dysthyroid optic neuropathy (DON), with the condition resolving in 66% of the patients.

Surprisingly, there is limited knowledge regarding the safety profile of this treatment. Hence, the aim of this study was to describe the occurrence of local and systemic adverse events after PT injections in patients with TED.

## METHODS

This retrospective case series was approved by the Institutional Review Board at the Hospital Italiano de Buenos Aires (Buenos Aires, Argentina) and adhered to the tenets of the Declaration of Helsinki. A database search of all patients with TED who received PT injections from 2007 to 2019 at Hospital Italiano de Buenos Aires was performed. The primary endpoint was the identification of local and systemic adverse effects. The secondary endpoint was the efficacy of PT alone in the treatment of orbital inflammation in TED, or the requirement for other treatments.

The inclusion criteria for patients were: 1) age >18 years; 2) recent diagnosis of TED (<6 months); and 3) treatment with PT. The criteria for the treatment of patients were: 1) Clinical Activity Score (CAS) ≥3; 2) moderate or severe disease (defined using the European Group on Graves’ orbitopathy [EUGOGO] criteria); and 3) mild disease and CAS <3 with intraorbital inflammation signs detected using orbital short tau inversion recovery-sequence magnetic resonance imaging (STIR-MRI). The exclusion criterion was previous treatment with systemic steroids, radiotherapy, or immunosuppressants 6 months prior to receiving the PT injection.

Treatment was performed through retroocular PT injection in the inferior lateral quadrant of the orbit. The entire 25-gage, one-inch, disposable needle was buried through the eyelid and 20 mg/0.5 ml PT was placed in each orbit weekly for a total of four applications. The injection was performed always in the same orbital location by five different oculoplastic surgeons and five different oculoplastic fellows. In the absence of clinical improvement, treatment was extended until the resolution of inflammation. For patients with a good response and appearance of recurrent inflammation, a new complete treatment cycle comprising four injections was administered. Systemic corticosteroids were administered to patients who experienced a deterioration in the severity of the orbitopathy (defined based on the EUGOGO criteria) despite local treatment.

To evaluate the presence of complications/adverse effects, we classified all events into local and systemic. Local complications were determined by the presence of at least one of the following effects after treatment: decreased visual acuity, diplopia worsening, increased intraocular pressure (IOP), cataract, ocular injury or perforation, eyelid edema or ecchymosis, retrobulbar hemorrhage, fat herniation, local corticoid dermopathy, and herpes reactivation. Systemic complications were determined by the presence of at least one of the following: increased weight, presence of Cushing, hypertension, hyperglycemia, adrenal insufficiency after treatment discontinuation, and renal insufficiency.

Pre-existing conditions (e.g., hypertension, diabetes, and smoking) were evaluated to determine potential associations with more frequent side effects. Furthermore, the CAS and severity of TED in each patient were described.

Data were compiled, and descriptive statistics were calculated using Stata software version 13.0 (StataCorp, College Station, TX, USA).

## RESULTS

The clinical histories of 123 patients with TED who underwent treatment with PT injections between 2007 and 2019 were evaluated. Patient characteristics are summarized in table 1. The average duration of the follow-up was 6 years.

**Table 1:** Patient demographics

Total patients	n=123
Age, years (mean ± SD)	45.9±13.7
Sex	
Female	74.7% (n92)
Pre-existing condition	
Hypertension	13.0% (n16)
Diabetes	4.0% (n5)
Smoker	38.1% (45)
CAS, median (IQR)	2 (<2-3)
Severity	
Mild	55.5% (n 60)
Moderate	28.7% (n 31)
Severe	13.8% (n 15)
Sight-threatening	4% (n 5)
No data	9,75% (n 12)

SD= standard deviation; CAS= clinical activity score; IQR= interquartile range.

The mean age of patients was 45.9 ± 13.7, and most of them were females (74.7%). A mean of 4.5 ± 2.0 injections (range: 1-12) were required to alleviate symptoms. Eleven patients required systemic treatment due to lack of improvement in the CAS score after injections (n=6) and DON progression (n=5). All received systemic cor­ticosteroids, and four of the five patients with DON progression also required urgent decompressive surgery.

The complications associated with this treatment are summarized in table 2. A total of 11 local complications (8.9%) were documented; the most frequent complication was the presence of superficial eyelid ecchymosis (nine patients; 7.3%), followed by diplopia worsening (two patients; 1.6%) and ocular hypertension (one patient; 0.8%). Among the 123 patients included in the study, only two developed systemic complications. One patient (with pre-existing diabetes) had uncontrolled hyperglycemia after four injections, and one patient experienced suprarenal inhibition after prolonged treatment (i.e., eight PT injections in each orbit). One week after completing the eight injections, the patient complained of fatigue, intense weakness, headache, and experienced an episode of syncope. We referred the patient to an endocrinologist, who reached the diagnosis of adrenal insufficiency based on the low basal levels of blood cortisol; the patient was treated with oral hydrocortisone.

**Table 2:** Complications associated with injection of peribulbar triamcinolone

Total patients	n=123
Local complications	**8.9% (n11)**
Ecchymosis	7.3% (n9)
Diplopia worsening	1.6% (n2)
Ocular hypertension	0.8% (n1)
Systemic complications	**1.6% (n2)**
Hyperglycemia	0.8% (n1)
Adrenal insufficiency	0.8% (n1)

## DISCUSSION

In this study, all adverse effects were transitory, and the patients did not have any long-term sequelae. Regarding local complications, all cases of eyelid ecchymosis were superficial and resolved spontaneously. Worsening of diplopia occurred only in two patients, who had constant diplopia and severe restrictive strabismus prior to receiving PT. In both cases, the patients reported discomfort because of a change in their vertical diplopia. Due of the temporal relationship between the injections and the modification in diplopia, we considered both effects as complications rather than indicators of TED progression. Triamcinolone may alter the volume of the extraocular muscles^(11)^ (detected through computed tomography after treatment) and, consequently, their function and excursion. This suggests that patients may need to readapt to a new coordination between both eyes in the binocular vision. In all patients, the IOP was measured at 1 week, 1 month, and 3 months after the administration of four PT injections. Ocular hypertension was reported in only one patient who exhibited a good response and control with topical anti-glaucoma medication. In this study, the incidence of IOP increase was markedly lower (0.8%) than that previously reported by Poonyathalang et al. in 2005^(13)^ (20.0%). This may be attributed to the difference in the dosage of PT per orbit between the two studies (20 mg vs. 40 mg, respectively). Although these investigators reported a high incidence of ocular hypertension, all their patients had good management and control with the use of eye drops and did not require surgery. In the study conducted by Bordaberry et al.^(12)^, the most frequent local adverse effect was increase in IOP (9.5%), and there were no systemic adverse effects recorded. Although the incidence was higher in the previous study versus our investigation, the former included a smaller number of patients (i.e., 21 patients) with more severe orbitopathy; this population may have included patients with more venous congestion.

Alkawas et al.^(14)^ revealed that local treatment with PT is also linked to systemic complications; 8% of patients had increased blood pressure and gastritis. However, the rate of occurrence was significantly lower compared with that noted for systemic corticosteroids (i.e., between 50% and 75% of patients developed these complications, In that study, the investigators did not report local complications.

In the present study, systemic complications were uncommon (one patient; 1.6%). One patient (with pre-existing diabetes) developed uncontrolled hyperglycemia after four injections and required additional insulin, finally reaching good metabolic control. Despite the very low rate of this complication, it is advisable to control blood pressure and glycemia after PT injections in patients with pre-existing conditions. Adrenal insufficiency is another very rare adverse effect (0.8%) that may occur in patients that require more than four injections. Ebner et al.^(11)^ described a decrease in urinary cortisol in 31.5% of treated patients, without reporting any case with clinic insufficiency that required treatment. Hence, in patients who require extended treatment, the monitoring of signs and symptoms of adrenal insufficiency (e.g., abdominal pain, confusion, unconsciousness, dehydration, vertigo or dizziness, fatigue, intense weakness, headache, high fever, or loss of appetite) is important. In the absence of clinical suspicion, laboratory testing is not required.

Ebner et al.^(11)^ described for the first time the benefits of PT in TED. Goldberg in 2004^(15)^ and Poonyathalang et al. in 2005^(13)^ confirmed these benefits and the low incidence rate of systemic adverse effects. In a prospective study conducted by Bordaberry et al.^(12)^ in 2009, a significant improvement in soft tissue edema was noted, thereby improving muscle function, diplopia, and proptosis. The investigators also reported that PT injections could reverse DON (resolution rate: 66%). In 2010, Alkawas et al.^(14)^ compared the efficacy of PT versus that of oral systemic corticosteroids. They concluded that PT reduces inflammation, thus improving eye movements, diplopia, and proptosis. In 2018, using nuclear magnetic resonance STIR, Ortiz-Basso et al.^(16)^ showed that the orbital anti-inflammatory benefit of PT can be observed directly in clinical improvement as well as indirectly in the reduction of orbital inflammation.

In our report, 108 patients with TED (87.8%) had good local control of the orbital and palpebral involvement of the disease (a decrease in the CAS score <3). This finding was consistent with the good response reported in previous studies. Eleven (8.5%), patients required other treatment owing to poor control of the orbitopathy, six due to lack of improvement in the CAS score after injections, and five because DON progression. All received systemic corticosteroids; and four of the five patients with DON progression also required urgent decompressive surgery. The preoperative clinical examination showed a decrease in visual acuity (1/4), relative afferent pupil defect (4/4), impairment in the visual field (3/4), and alteration in the color Farnsworth test (3/4). All postoperative controls demonstrated an improvement in their visual field and color test, and the patient with decreased vision restored his vision (10/10) in both eyes. Only one patient (0.8%) discontinued the treatment due to a superficial eyelid ecchymosis, which developed after the first injection.

Currently, it is unclear whether patients with TED with a CAS <3, who have one or two clinical signs of inflammation are actually inactive. Because of the presence of inflammation signs, we think that these patients are active. Through orbital STIR-MRI, we can detect orbital inflammation in patients with CAS <3 and administer anti-inflammatory treatments accordingly^(16)^. Currently, the focus of therapy in this setting is on control and the administration of eyedrops. Therefore, PT injections may be an effective option for treating and improving the conditions of these patients.

The main limitation in this study is inherent in its design. This was a retrospective case series, and data were collected from the electronic medical history records of patients. Therefore, it is possible that complications may have been overlooked by different physicians.

Importantly, this was the largest case series investigating PT injections in TED, and the first study to assess its safety profile. The rate of local complications in this study was extremely low, and all events were transitory without consequences in the vision of patients. Systemic complications are unusual and should be considered in patients with previous comorbidities or undergoing prolonged treatment (i.e., more than four injections). Prospective studies are warranted to delve into this topic.
